# A Low-Frequency Vibration Sensor Based on Ball Triboelectric Nanogenerator for Marine Pipeline Condition Monitoring

**DOI:** 10.3390/s24123817

**Published:** 2024-06-13

**Authors:** Xili Huang, Bin Wei, Ziyun Ling, Fang Yang, Hongchen Pang

**Affiliations:** 1 Naval Architecture and Shipping College, Guangdong Ocean University, Zhanjiang 524088, China; 2112010010@stu.gdou.edu.cn (X.H.); 2112106011@stu.gdou.edu.cn (Z.L.); 2 Guangdong Provincial Key Laboratory of Intelligent Equipment for South China Sea Marine Ranching, Guangdong Ocean University, Zhanjiang 524088, China; shipin@gdou.edu.cn (B.W.); neomailyf@gdou.edu.cn (F.Y.); 3 School of Food Science and Technology, Guangdong Ocean University, Zhanjiang 524088, China; 4 School of Mechanical Engineering, Guangdong Engineering Technology Research Center of Ocean Equipment and Manufacturing, Guangdong Ocean University, Zhanjiang 524088, China

**Keywords:** triboelectric nanogenerator, condition monitoring, vibration sensor, low frequency

## Abstract

Marine pipeline vibration condition monitoring is a critical and challenging issue, on account of the complex marine environment, while powering the required monitoring sensors remains problematic. This study introduces a vibration sensor based on a ball triboelectric nanogenerator (B-TENG) for marine pipelines condition monitoring. The B-TENG consists of an acrylic cube, polyester rope, aluminum electrodes, and PTFE ball, which converts vibration signals into electrical signals without the need for an external energy supply. The experimental results show that B-TENG can accurately monitor the frequency, amplitude, and direction of vibration in the range of 1–5 Hz with a small error of 0.67%, 4.4%, and 5%, and an accuracy of 0.1 Hz, 0.97 V/mm, and 1.5°, respectively. The hermetically sealed B-TENG can monitor vibration in underwater environments. Therefore, the B-TENG can be used as a cost-effective, self-powered, highly accurate vibration sensor for marine pipeline monitoring.

## 1. Introduction

The ocean is a treasure house of rich and diverse resources: it is rich in oil, natural gas, metal minerals and other precious resources. With the increasing demand of human society for the development of marine resources, a series of key marine structures, such as cages, risers, and submarine pipelines, have been widely deployed in various seas around the world in recent years [1]. Among them, marine pipelines not only integrate the functions of resource collection, transmission, and support but also constitute the core component of the development and utilization system of marine resources. They also play a pivotal role in ensuring the safety of offshore operations and the stable operation of production systems [2]. However, the complex natural dynamic factors in the marine environment, such as the dynamic load caused by waves and currents, make underwater pipelines prone to strong and nonlinear dynamic response, which poses a significant threat to the structural integrity and operational safety of pipelines [3,4,5,6,7]. To ensure the production safety in the process of marine resources development, it is very important to accurately obtain the real-time status information of underwater pipelines. Using advanced vibration monitoring technology, real-time monitoring of pipeline vibration and in-depth data analysis can be sensitive to identify the abnormal vibration conditions or potential structural damage of pipelines. And timely preventive intervention measures or emergency maintenance can be taken to effectively avoid potential safety accidents and ensure the continuity and safety of operation activities. At present, sensors applied to the vibration monitoring of marine pipeline mainly include resistance strain gauge sensors [8,9,10], acoustic sensors [11,12,13,14] and optical fiber sensors [15,16,17,18]. Although they show certain advantages in monitoring accuracy and reliability, their common limitation is that they all rely on traditional wire power supply or built-in battery power supply. Firstly, the high construction costs and technical difficulties of constructing submarine cable networks as power supply infrastructure, especially in deep-sea areas far from the continental shelf, cannot be ignored. In addition, the unavoidable energy loss problem during long-distance power transmission further weakens the economy and effectiveness of this power supply method [19]. Secondly, although the battery can provide certain life support as a backup power supply, its service life is limited, and it needs to be replaced after the power is exhausted. This process significantly increases the cost of operation and maintenance but also may lead to a temporary interruption of monitoring work, affecting the continuity and accuracy of data collection [20]. In addition, vibration direction monitoring can help engineers and operators understand the main direction and source of vibration, so that corresponding measures can be taken to reduce or eliminate these vibrations. However, these vibration sensors mainly focus on vibration frequency or amplitude monitoring, and they pay less attention to vibration direction detection. In view of the limitations of the current marine pipeline vibration monitoring sensors in the power supply mode, it is particularly urgent and significant to develop new self-powered vibration sensors in the face of the long-term and reliable state monitoring requirements of marine pipelines.

**Figure 1:**
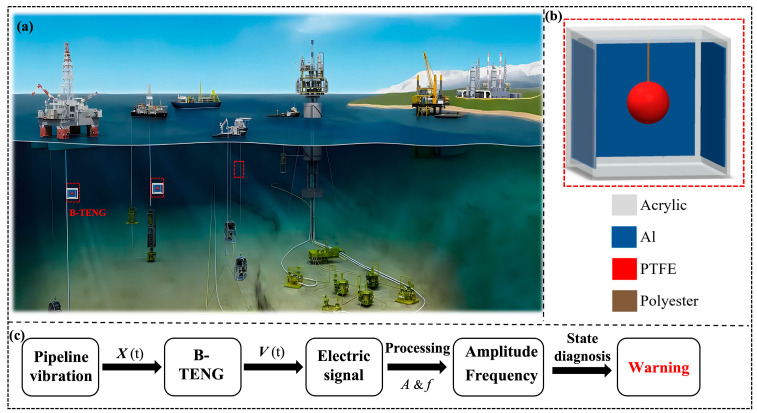
Application scenario and structure of B-TENG sensor. (**a**) Schematic diagram of B-TENG applied to marine pipeline vibration monitoring; (**b**) structure of B-TENG; (**c**) the flow chart of B-TENG for detecting pipeline conditions.

The triboelectric nanogenerator (TENG) was first proposed by Wang et al. in 2012 [21]. TENG is an innovative device that converts mechanical energy into electrical energy through contact electrification and electrostatic induction [22,23,24,25,26,27]. TENG can be used not only as a self-driven sensor to sense various mechanical and physical signals but also to harvest ambient mechanical energy, integrating energy storage and management to realize a self-charging power supply system to provide continuous power to other small electronic or communication devices. Its potential applications in energy harvesting and self-powered sensors have attracted great attention [28,29]. Numerous studies have shown that TENG has become a versatile and efficient energy-harvesting and self-powered sensing technology with applications ranging from small-scale portable electronics to large-scale blue energy harvesting [30,31,32,33,34]. TENG has the advantages of low cost, light weight, simple structure, strong environmental compatibility, wide material selection, etc., showing obvious advantages in the field of self-powered vibration sensing technology [35,36,37,38,39,40,41]. In this paper, a vibration sensor based on ball TENG (B-TENG) was designed for marine pipelines condition monitoring. The arrangement of the B-TENG sensors for monitoring vibrations in marine pipelines is shown in Figure 1a, which includes flexible risers and submarine pipelines. The B-TENG consists of an acrylic cube, a polyester rope, aluminum electrodes, and a PTFE ball, as shown in Figure 1b. The B-TENG generates an electrical output when the pipe vibrates. The electrical output of the B-TENG is measured by the electrometer, which sends the signals to the computer. After signal processing, the signals reflecting the actual vibration output by the B-TENG can be obtained to determine the vibration status of the pipe, as shown in Figure 1c. The experimental results show that B-TENG can detect the direction, frequency, and amplitude of vibration in the range of 1–5 Hz, which has a broad application prospect in the field of marine pipeline vibration monitoring. The vibration sensors demonstrated in the literature and proposed B-TENG are compared as shown in Table 1.

## 2. Materials and Methods

### 2.1. Working Principle and Theoretical Model of the B-TENG Sensor

Based on the working principle of the freestanding layer mode, a vibration sensor based on ball TENG (B-TENG) was proposed. The B-TENG was made of an acrylic cube which was designed as a detachable structure for easy experimental operation. Four pieces of aluminum foil (Al) tape were pasted on the side wall of the inner surface of the acrylic housing as the electrode of the B-TENG. A ball was suspended from the center of the cube using a polyester rope, and the ball serves as the friction material for the B-TENG.

**Figure 2:**
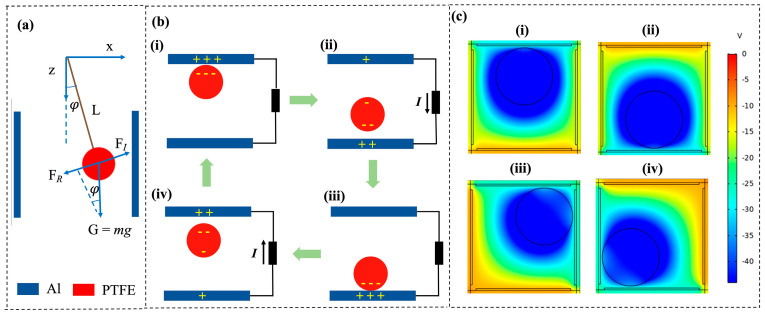
Working principle and simulation of the B-TENG. (**a**) Schematic of dynamic analysis of the ball oscillation in the cube; (**b**) working principle of the B-TENG in top view; (i) the ball is in contact with the top electrode. (ii) ball leaves top electrode. (iii) the ball is in contact with the bottom electrode. (iv) the ball leaves the bottom electrode and approaches the top electrode. (**c**) the electric potential distribution on the B-TENG sensor. Potential distribution as the ball approaches (i) the top electrode and (ii) the bottom electrode; Potential distribution as the ball moves along 45° angle to (iii) the top electrode and (iv) the bottom electrode.

The motion of the ball belongs to the secondary transmission; when the pipeline vibrates on the acrylic cube, the cube will produce the corresponding transverse rocking, transverse oscillation, and other relative movements. The forced vibration of the ball is mainly from the cube on the pipeline vibration excitation made by the response. As shown in Figure 2a, when the B-TENG is unaffected by external vibrations, the inertial force *F*_I_ (mass times acceleration) must be equal to the applied action force on the object. In B-TENG, the acting force is the restoring force *F*_R_, which is caused by the gravitational force *G*. To obtain the dynamic and electrical model of the B-TENG in non-contact mode, we assume that the amplitude and frequency of the cube excited by the vibration are A and *f*, respectively. The forced vibration law of the ball can be viewed as a damped resonant subsystem (approximately). The ball moves periodically and follows the dynamic equation:

(1) $$\ddot{\varphi }{-C}_{w}6\pi \mu r\dot{\varphi }+{\left(\omega_{0}\right)}^{2}sin\varphi ={\left(2\pi f\right)}^{2}\frac{A}{L}cos\left(2\pi ft\right)cos\varphi \;$$

where φ is the angle at which the ball swings (angular displacement), C_w_ is the wall correct factor, μ is the kinetic viscosity of the air, *r* is the radius of the ball, $\dot{\varphi }\;$is the angular velocity of the ball with respect to the fluid, $\omega_{0}$ is the rotational angular velocity of the inertial ball, and *L* is the pendulum length, as shown in Figure 2a. The detailed derivation of Equation (1) is shown in Figure S1 (Supporting Information).

According to the freestanding layer TENG theory, the total negative charge of the ball is ideally equal to the positive charge of the two electrodes. The governing equation of B-TENG can be written as [45]

(2) $$V=-\frac{1}{C}Q+V_{OC}=-\frac{d_{0}+D}{\varepsilon_{0}s}Q+\frac{2\sigma x}{\varepsilon_{0}}$$

where *C*, *Q*, *V_OC_*, *d*_0_, *D*, $\varepsilon_{0}$, *S*, *σ*, and *x* are the capacitance, transferred charge, open circuit voltage, effective dielectric medium thickness, total air-gap thickness between the two electrodes, effective contact area of the aluminum electrode layer, dielectric constant in vacuum, charge density on the surface of the ball, and distance between the electrode layer and the ball, respectively. The detailed derivation of Equation (2) is shown in Figure S2. Under the minimum achievable charge reference state (MACRS), the short circuit transferred charges (*Q*) and open-circuit voltage (*V_OC_*) can be calculated as follows:

(3) $$Q=\frac{2\sigma x}{d_{0}+D}\;$$

(4) $$V_{OC}=\frac{2\sigma x}{\varepsilon_{0}}\;$$

In fact, *x* is the displacement of the ball’s motion movement. The motion state of the ball is related to the state of external excitation. Therefore, analyzing the electrical signal of the B-TENG can determine the external vibration state.

The working principle is illustrated by the unidirectional movement of the ball along the *X*-axis in B-TENG. As shown in Figure 2b, in the absence of external excitation, the ball is placed in the center of the acrylic box, and no charge transfer is present. When vibratory excitation is applied externally, the ball begins to wobble. When the excitation strength reaches a certain level, the ball contacts the aluminum electrode. Due to the different abilities of the two materials to gain and lose electrons, the surface of the aluminum electrode and the ball carry an equal number of positive and negative charges, as shown in Figure 2b(i). After the ball is separated from the top electrode, a potential difference is generated between the two electrodes. To balance the potential difference, electrons in the external circuit flow from the bottom electrode to the top electrode, thus making the external circuit output electrical signals (Figure 2b(ii)). When the ball contacts the bottom electrode (Figure 2b(iii)), all the positive charges appear on the bottom electrode, and when the ball moves to the top electrons again, the electrons return, creating a reverse current in the outer circuit (Figure 2b(iv)). PTFE is an electric material in which the friction charge on the surface persists for a long time. Thus, the movement of PTFE between two electrodes causes a potential difference due to electrostatic induction, even if no physical contact exists between the PTFE and the aluminum electrode in the later working stages, and an electrical signal is generated. Therefore, the as-proposed B-TENG can accurately detect external vibrations in non-contact mode.

**Figure 3:**
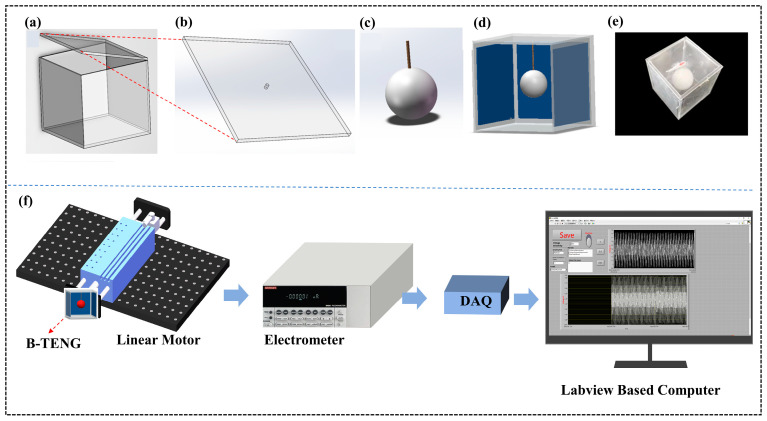
Schematic of B-TENG manufacturing and experimental platform. (**a**) Acrylic shell; (**b**) removable upper plate; (**c**) the ball; (**d**) schematic diagram of B-TENG; (**e**) physical diagram of B-TENG; (**f**) experimental setup of the B-TENG sensor.

COMSOL Multiphysics software was used to simulate the induced potential distribution of B-TENG under open circuit conditions, and the change process of the electrostatic field was explained by locating charged pellets at different positions, as shown in Figure 3c. In the initial state, the electrode and the surface of the ball are set to a uniform distribution of positive and negative charges, respectively. As shown in Figure 2c(i), when the ball moves upward, the distance between the ball and the top electrode decreases, resulting in a larger electric potential induced by the top electrode compared to the lower electrode. The ball then moves down to contact the bottom electrode (Figure 2c(ii)), generating the opposite induced potentials. Similarly, Figure 2c(iii,iv) are the changes in the electrostatic field as the ball moves to different positions along 45°. The potential difference generated by the periodic swing of the ball drives the electrons to flow back and forth in the external circuit, causing an alternating current to be generated in the external circuit. The simulation results are consistent with the above working principle.

### 2.2. Fabrication and Experimental Setup of the B-TENG Sensor

B-TENG consists of four main components.

(1) Acrylic housing (Nanchang Intech Trading Co., Ltd., Nanchang, China). To make it easier to observe the interior, a highly transparent acrylic sheet is used as the external housing of the B-TENG. Its thickness is 2 mm, and the internal dimensions of the square shell are 60 mm × 60 mm. It is worth noting that the use of acrylic as the shell of the B-TENG in this paper is mainly for the convenience of observing the vibration state of the ball in the cube, and other more suitable materials should be selected in practice for the sake of pressure resistance and sealing considerations.
(2) Electrodes. Four square aluminum foil (Shenzhen Milleqi Tape Co., Ltd., Shenzhen, China) tapes with an area of 55 mm × 55 mm and a thickness of 0.2 mm were selected, which could be directly adhered to the inner wall of the acrylic housing. At the same time, enameled copper wires were used to connect the electrodes to the data measurement end of the electrometer.
(3) Dielectric balls (Yiwu Hongzhou Trading Co., Ltd., Jinhua, China) The different balls (diameter of 30 mm) are made of PP, PA, PTFE, and POM, respectively. The selected materials have good abrasion resistance. A 1 mm diameter hole is punched in the center of the ball for easy hanging.
(4) Polyester rope (Wenzhou Nazhi Fasteners Co., Ltd., Wenzhou, China). Polyester rope with a diameter of 0.2 mm was chosen because of its low modulus of elasticity; even if it is subjected to prolonged stress in the experiment, the deformation is very small, and it is suitable for hanging the ball in the cube.

First, as shown in Figure 3a, the upper cover of the acrylic shell was designed as a detachable structure for convenient experimental operation; As shown in Figure 3b, a small 1.5 mm hole was cut in the center of the top cover for hanging the inner ball and adjusting the length of the polyester rope. As shown in Figure 3c, the polyester rope was connected to the center of the ball by punching holes. Finally, the four aluminum foil electrodes were fixed to the inner wall of the acrylic plate, and the ball was suspended to the exact center of the box along the small hole in the top cover. The schematic diagram of the B-TENG assembly is shown in Figure 3d. The electrode was connected to the electrical signal acquisition terminal by a copper wire for recording experimental data. The final physical diagram of B-TENG is shown in Figure 3e.

Figure 3f shows the experimental system for the B-TENG performance test. The test system consists of a linear motor, an electrometer, data acquisition equipment, and the LabVIEW software. The linear motor is used to output vibration excitation at low frequencies (1–5 Hz) and different amplitudes (2–14 mm). First, the linear motor was fixed on the iron plate to ensure it was in a horizontal position, and the B-TENG device was fixed on the working end of the linear electrode. The opposite aluminum electrode consisted of a set of metal electrodes, and the leading wire at the electrode was connected to the positive and negative measuring ends of the electrometer. Then, the electrical signal data measured by the electrometer were converted into a digital signal through the NI data acquisition card and transmitted to the computer. The data were displayed and stored on the computer using LabVIEW software (Version 2018, National Instruments, Cleveland, OH, USA), and the program set by the software could realize a sampling frequency of 500 Hz. Meanwhile, to measure the relationship between the output electrical signals of B-TENG at different angles, two 30° and 45° structural parts were designed using SOLIDWORKS software (Version 2019, Waltham, MA, USA). These parts were manufactured by a 3D printer and fixed to the working end of the linear motor to realize the subsequent quantitative test experiment of vibration angle. In our experiments, the linear motor parameters were first set to generate vibrations with a frequency of 3 Hz and an amplitude of 10 mm to bring the ball into contact with the aluminum electrode. When the voltage of B-TENG reaches the maximum value and stabilizes, the surface charge density of the ball was saturated currently. After that, the voltage of B-TENG in non-contact mode was measured in the frequency range of 1–5 Hz and the amplitude range of 2–14 mm.

## 3. Results and Discussion

### 3.1. Influence of Structural Parameters on B-TENG Output Performance

**Figure 4:**
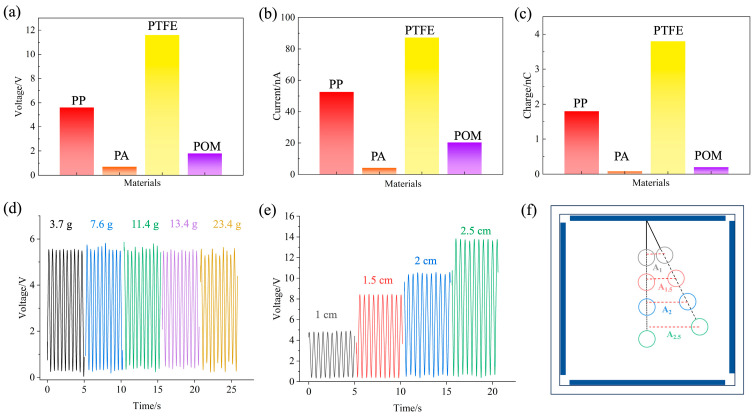
Influence of structural parameters on the output performance of B-TENG. (**a**) Voltage, (**b**) current, (**c**) charge of different ball materials B-TENG under the vibration with frequency of 2 Hz and amplitude of 14 mm; (**d**) output voltage of B-TENG with different ball mass; (**e**) output voltage of B-TENG with different rope lengths at 2 Hz and 10 mm; (**f**) schematic diagram of rope length affecting amplitude.

The selection of friction materials is crucial to improve the performance of triboelectric nanogenerators. The only available tool that describes the triboelectrification of materials is a triboelectric series [46]. According to the triboelectric series shown in Figure S3, considering cost and accessibility, a variety of common friction materials such as polyformaldehyde (POM), polypropylene (PP), nylon (PA), and polytetrafluoroethylene (PTFE) were selected for comparative analysis. Figure 4a–c show the output voltage, short-circuit current, and transferred charge of the B-TENG at a vibration frequency of 2 Hz and an amplitude of 14 mm, respectively. It is shown that when PTFE was used as the ball material, the output voltage, short-circuit current, and transferred charge of the B-TENG reached the maximum. This is because POM, PP, PA, and PTFE are at different positions in the friction charge sequence. In contact with the aluminum electrode, PTFE lost electrons more readily than the other three, so the more friction charges the ball carried, the stronger the output performance. Meanwhile, because PA material and aluminum are the closest in the quantified triboelectric serials, the amount of friction charge generated was small, and the output electrical signal was the weakest. According to the experimental results, the ball material of B-TENG was made of PTFE, which could generate larger electrical signals, effectively reduce the influence of external free charge on the output of the device and improve the signal-to-noise ratio of the device. In addition, because PTFE is an electric material, the surface charge can be maintained for a long time. After that, even if the electrode is not in contact, a strong electrical signal output can still be generated between the PTFE ball and the electrode due to the principle of electrostatic induction. Therefore, in the subsequent experiments, PTFE was chosen as the friction layer material for further study.

The output voltage of B-TENG with the same diameter (30 mm) and different mass of PTFE ball was measured with a rope length of 2 cm at the external amplitude of 10 mm and frequency of 2 Hz. As shown in Figure 4d, the results indicated that the output voltage of B-TENG was essentially the same when the mass of the PTFE ball was 3.7 g, 7.6 g, 11.4 g, 13.4 g, and 23.4 g, respectively. The mass of the PTFE ball did not influence on the output of B-TENG. The output voltage of B-TENG for polyester rope lengths of 10, 15, 20, and 25 mm was measured with a ball mass of 23.4 g at a vibration frequency of 2 Hz and an amplitude of 10 mm. As shown in Figure 4e, the output voltage of the B-TENG showed a gradual increase as the length of the polyester rope was increased. Data analysis through fitting revealed a linear relationship between the output voltage of the B-TENG and the polyester rope length with an exceptionally high linear correlation coefficient (R^2^) of approximately 0.994. Due to the inertia effect, the PTFE ball can synchronously move at the same frequency with the linear motor, but the vibration amplitude of the PTFE ball depends on the length of the rope. As shown in Figure 4f, under the same vibration excitation, extending the rope length leads to an increase in the amplitude (A) of the PTFE ball’s motion. As shown in Figure 4f, A_1_, A_1.5_, A_2_, and A_2.5_ are the amplitude of the swing of the ball when the length of the string is 1 cm, 1.5 cm, 2 cm, and 2.5 cm, respectively. Under the same vibration excitation, extending the rope length led to an increase in the amplitude (A) of the PTFE ball’s motion. This amplification in amplitude brought the charged PTFE ball closer to the electrodes on either side, consequently augmenting the output electrical signal. Therefore, the design of the B-TENG should be noted: after the external housing size is determined, increasing the length of the rope increases the voltage output of the device, but the problem is that the increase in amplitude will make it easier for the PTFE ball to contact the electrodes on both sides of the B-TENG, so that the measurement bandwidth of the B-TENG device will be reduced. In addition, the length of the rope determines the resonance frequency of the system; specifically, the length of the rope is inversely proportional to the resonance frequency. Therefore, it is necessary to select the appropriate rope length according to the vibration characteristics of the measured target. Based on the above experimental results, the friction layer material, ball mass, and rope length are optimized to improve the electrical output performance of the device. Based on the above experimental results, B-TENG with a PTFE ball of 23.4 g and a rope length of 2 cm was selected for subsequent experiments.

### 3.2. Effect of Vibration Parameters on B-TENG Output Performance

**Figure 5:**
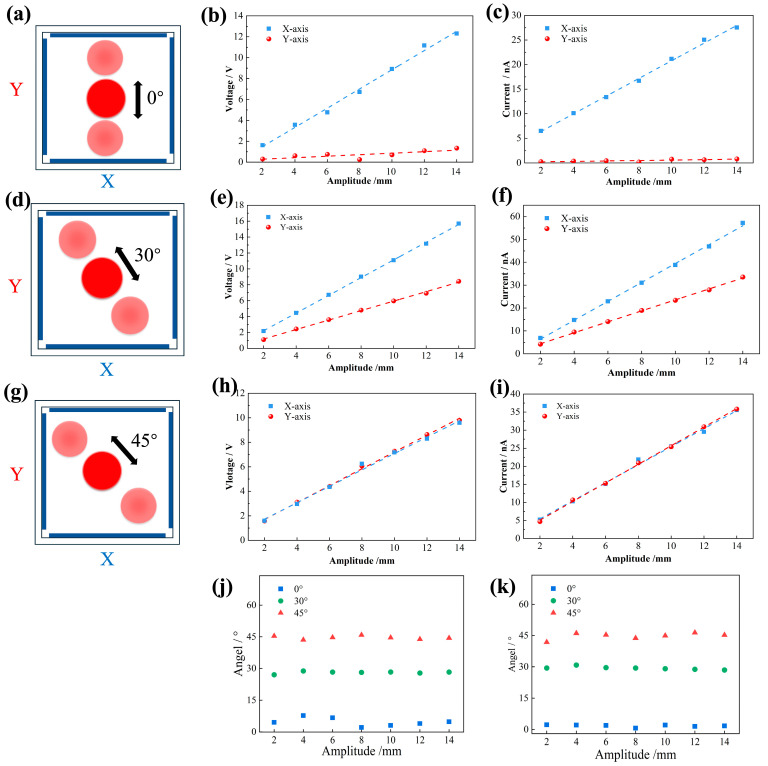
Influence of vibration direction on the output performance of B-TENG at different amplitudes with a fixed frequency of 2 Hz. (**a**) Schematic diagram in top view when the vibration angle is 0°; (**b**) relationship between voltage and amplitude; (**c**) relationship between current and amplitude; (**d**) schematic diagram in top view when the vibration angle is 30°; (**e**) relationship between voltage and amplitude; (**f**) relationship between current and amplitude; (**g**) schematic diagram in top view when the vibration angle is 45°; (**h**) relationship between voltage and amplitude; (**i**) relationship between current and amplitude; the angle calculated from the B-TENG (**j**) voltage; (**k**) current.

The direction of vibration reflects the instantaneous direction of movement of the object, and judging the direction of movement of the vibrating object can effectively reflect the force situation of the object, which has an important role in analyzing the stress fatigue of the object. Theoretically, the output electrical signal of B-TENG in the *X*- and *Y*-axis directions should meet the following formula:

(5) $$tan\theta =\frac{Y_{\left(V,I\right)}}{X_{\left(V,I\right)}}$$

where *θ* is the vibration angle, *Y_(V,I)_* is the output electrical signal between the electrodes in the *Y*-axis direction, and *X_(V,I)_* is the output electrical signal between the electrodes in the *X*-axis direction. To verify that B-TENG can measure vibration in multiple directions, the characteristic quantity of B-TENG’s output electrical signal was studied systematically. The B-TENG was fixed to the working end of the linear motor by using 3D-printed structural parts, which were, respectively, 0°, 30°, and 45°. B-TENG could realize vibration in different directions by using structural components designed at different angles. The output electrical signals of B-TENG oscillating at 0°, 30°, and 45° were measured. Figure 5a shows the schematic diagram in top view of the ball moving along the *Y*-axis at an angle of 0°. Figure 5b shows the output voltages in *X*- and *Y*-axis directions when the angle is 0° and the vibration frequency is 2 Hz, and Figure 5c shows the output currents. Clearly, the output voltage and current amplitude of the *X*-axis maintained a good linear relationship with the vibration amplitude. A very small output electrical signal was also observed in the *Y*-axis direction. According to the simulation results shown in Figure 2c(i,ii), when the PTFE ball moves in the direction of 0°, it is perpendicular to the *Y*-axis; at this time, the PTFE ball has no relative displacement with the electrodes on both sides of the *Y*-axis, and there is no change in relative potential, so the output electrical signal should be 0. This error was caused by the deviation of the ball hanging in the center of the box body when the B-TENG experimental device was manufactured by hand. When the ball moves along the *X*-axis direction, a small displacement occurred in the *Y*-axis direction, which resulted in a small electrical signal output by the *Y*-axis electrode. Figure 5d shows the schematic diagram in the top view of the ball moving along the *Y*-axis at an angle of 30°. When the vibration direction is 30° and 45° along the *X*- and *Y*-axis and the vibration frequency is 2 Hz, the output electrical signals with different amplitudes were measured. As shown in Figure 5d, when the angle is 30°, according to Formula (2), the peak signal of voltage should satisfy the equation: *X_(V,I)_* = $\sqrt{3}$ *Y_(V,I)_*. Figure 5e,f show the relationship between the output voltage and current of B-TENG and the amplitude in the X and Y directions, respectively. Both have a good linear relationship with the amplitude. Figure 5g shows the schematic diagram of the ball moving along the *Y*-axis at an angle of 45°. In this state, the equation is satisfied: *X_(V,I)_* = *Y_(V,I)_*. As shown in Figure 5h,i, the output voltage and current of the *X*-axis was basically the same as that of the *Y*-axis, which is consistent with the simulation results shown in Figure 2c(iii,iv). Meanwhile, the vibration angles (*θ*) with different amplitudes are calculated according to Equation (5). Figure 5j,k show the vibration angle (*θ*) calculated according to the output voltage and current, respectively. When the amplitude is 2–14 mm, the vibration angle (*θ*) maintains good accuracy at 0°, 30°, and 45°, and the average error is less than 5%. The resolution of B-TENG for angle monitoring is about 1.5°. The poor resolution for angle detection is due to errors caused by production.

**Figure 6:**
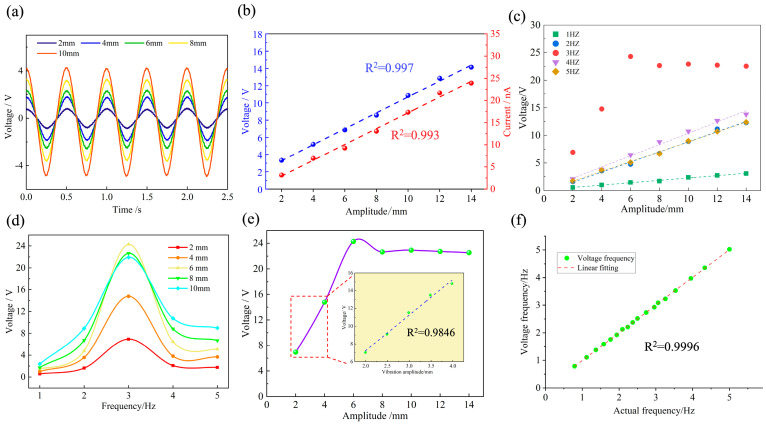
Influence of vibration parameters on the output performance of B-TENG. (**a**) B-TENG voltage output at different amplitudes of 2 Hz; (**b**) relationship between peak value of output voltage and current and amplitude; (**c**) relationship between amplitude and output voltage at different frequencies; (**d**) output voltage of B-TENG at different amplitudes of 1–5 Hz; (**e**) output voltage of B-TENG at different amplitudes of 3 Hz; (**f**) FFT results of the actual vibration frequency and voltage signals.

As the most basic vibration parameter, vibration amplitude is indispensable in measuring the vibration state of the structure. In mechanical vibration, the amplitude usually refers to the maximum displacement caused by an object vibrating away from its equilibrium position, and the amplitude is numerically equal to the absolute value of the maximum displacement size. It is used to describe the magnitude and strength of an object’s vibration amplitude. The low-frequency vibration (1–5 Hz) was mainly investigated, and the relationship between the amplitude and the output voltage of B-TENG was analyzed. First, the B-TENG was fixed to the working end of the linear motor, and the linear motor was set to run in a sinusoidal mode with a vibration frequency of 2 Hz and an amplitude of 2 mm, 4 mm, 6 mm, 8 mm, and 10 mm, respectively. As shown in Figure 6a, B-TENG also outputted voltage signals with sinusoidal waveform at the same frequency. Meanwhile, the output voltage of B-TENG also increased as the external amplitude gradually increased. The corresponding relationship between vibration amplitude and output voltage and current is further established, and it is clearly seen that there was a linear relationship between them, as shown in Figure 6b. Using voltage as a reference, the B-TENG measures amplitude at 2 Hz with a sensitivity of about 0.97 V/mm, a minimum resolution of 0.1 mm, and an average error of about 4.4%.

Subsequently, the relationship between the output voltage and amplitude of B-TENG at different frequencies (1–5 Hz) was measured experimentally, and the results are shown in Figure 6c. When the vibration frequency is between 1 Hz, 2 Hz, 4 Hz and 5 Hz, the B-TENG output voltage had a linear relationship with the amplitude. Specifically, the output voltage of the B-TENG was proportional to the amplitude at the same frequency. Linear fitting was performed on the data, and the fitting degree (R^2^) between them was 0.9927 (1 Hz), 0.9936 (2 Hz), 0.9907 (4 Hz), and 0.9957 (5 Hz), respectively. When the vibration frequency is 3 Hz, the output voltage of B-TENG was different from other frequencies. The relationship between the output voltage of the B-TENG, and the vibration frequency at the same amplitude continued to be analyzed. As shown in Figure 6d, the output voltage of B-TENG with a frequency of 3 Hz was always the largest when the amplitude was the same, indicating that the amplitude of the PTFE ball inside the cube was the largest. It was found that under vibration conditions at a frequency of 3 Hz, the voltage of the B-TENG increased linearly with respect to the external amplitude in the range of 2 to 6 mm and reached a maximum at 6 mm (the ball was already in contact with the aluminum electrodes at an amplitude of 6 mm). As the external amplitude continued to increase from 6 to 14 mm, there was no increase in the amplitude of the ball motion, so the voltage output of the B-TENG did not change significantly. According to Equation (6), the resonant frequency $f_{0}$ for the PTFE ball in the cube (with a rope length of 2 cm) is obtained to be about 3.04 Hz.

(6) $$f_{0}=\frac{1}{2\pi }\sqrt{\frac{g}{L}}\;$$

Due to the resonance phenomenon, the vibration amplitude of the PTFE balls in the cube increased significantly at 3 Hz, producing an output voltage that is different from other frequencies.

Due to the special performance of the B-TENG during vibration at a frequency of 3 Hz, the amplitude measurements were continued to be refined with amplitudes ranging from 2 to 4 mm at 0.5 mm intervals. The relationship between the output voltage of the B-TENG and the external amplitude was measured and analyzed mainly when the PTFE ball did not come in contact with the aluminum electrodes (non-contact mode). As shown in Figure 6e, the output voltage of B-TENG still maintained a linear relationship with the amplitude, and the fitting degree is about 0.9846. Therefore, at low-frequency vibration (1–5 Hz), the external vibration amplitude can be measured accurately based on the output voltage of B-TENG in non-contact mode.

Frequency is another important parameter of vibration. The relationship between the frequency of the B-TENG voltage signal and the vibration frequency was measured by varying the vibration frequency of the linear motor at the same vibration amplitude. As shown in Figure 6f, in the range of 1 to 5 Hz, the frequency of the voltage signal after FFT processing has a good linear relationship with the vibration frequency of the linear motor with a correlation coefficient R^2^ of 0.99. The average error is about 0.67%, and the minimum resolution could reach 0.1 Hz. Additionally, the average error is about 0.67%, and the minimum resolution could reach 0.1 Hz, demonstrating excellent vibration main frequency monitoring characteristics. Therefore, the voltage signals of B-TENG can be used to obtain the real-time vibration main frequency of marine pipelines via fast Fourier transform analysis to monitor their vibration status.

### 3.3. Comparative Analysis of B-TENG Vibration Response Experiments

**Figure 7:**
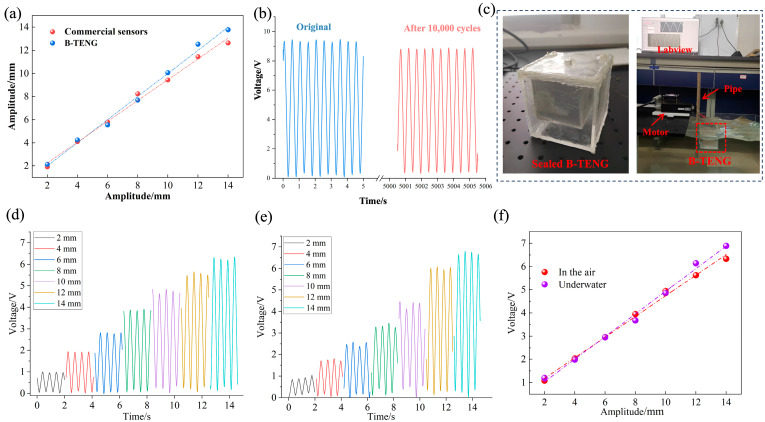
Application verification analysis of B-TENG. (**a**) B-TENG measured amplitude results compared to commercial sensors; (**b**) durability test results; (**c**) sealed B-TENG and underwater test platform; output voltage of B-TENG at different amplitudes with a fixed frequency of 2 Hz; (**d**) in air; (**e**) underwater; (**f**) comparison between them.

At a vibration frequency of 2 Hz, when the external vibration amplitude changes from 2 to 14 mm, the vibration amplitude measured by the B-TENG is highly consistent with that of the commercial sensor, as shown in Figure 7a. The stability of B-TENG was tested. The output voltage of the B-TENG was measured after 10,000 operating cycles with no contact between the PTFE balls and the aluminum electrodes on either side. As shown in Figure 7b, there was a slight decrease in the output voltage of the B-TENG after 10,000 cycles, which was attributed to the natural loss of surface charge of the PTFE ball. In the air, due to environmental factors (such as humidity, ion concentration, etc.), the accumulated charge on the surface of the ball gradually decreases and cannot be maintained in a saturated state, resulting in weakened device performance. If the inside of the B-TENG device is pumped to a vacuum state, the factors that cause charge loss in the air are eliminated. In a vacuum, there is no charge carrier such as water and ions, and the natural loss in surface charge of the PTFE ball is significantly reduced, which can maintain a high charge density for a long time, ensuring that the B-TENG still has good stability in the absence of contact. In addition, if the PTFE ball can contact the electrode within a certain period, this contact can accumulate charge by contact electrification, thus maintaining the saturation state of the surface charge of the ball and ensuring the stability of the output voltage. Because the underwater environment is different from the air environment, there are many free electrons in water, which have a great impact on the output of B-TENG. To verify the performance of B-TENG under water, as shown in Figure 7c, the B-TENG was enclosed in the shell so that its shell did not have direct contact with water. The pipe was fixed to the working end of the linear motor, and the B-TENG was fixed to the pipe. The linear motor provided simulated vibration, and the voltage signal of the B-TENG in the underwater environment was measured and compared with the measurement results in the air environment at different amplitudes with a fixed frequency of 2 Hz. Figure 7d,e show the output voltage signals of B-TENG in air and underwater, respectively. The output voltage signal of B-TENG in water was not significantly different from that in air. After that, the peak values of voltage in water and air were extracted, respectively, for fitting, and the results are shown in Figure 7f. The measurement data in underwater and air were basically consistent. The results for air and water differed only at amplitudes of 12–14 mm, mainly in the form of slightly smaller voltages underwater than in air. This is because larger water waves are generated when the amplitude of the linear motor is large enough, and the homemade pool is not long enough to dissipate the waves, causing them to reflect off the end of the pool. This reflected water wave acted on the B-TENG to reduce the relative displacement of the ball, resulting in a slight drop in the peak voltage. It is proved that B-TENG monitors the vibration status of structures in underwater environments.

## 4. Conclusions

Based on the freestanding layer triboelectric nanogenerator principle, a self-powered vibration sensor for monitoring the marine pipeline is proposed and studied. The B-TENG sensor displayed a high sensitivity and strong stability. The external vibration state can be converted into an electrical signal by B-TENG without an external energy supply. The output electrical signal characteristics of the B-TENG under different excitation amplitudes, frequencies, and vibration directions were investigated. The conclusions are as follows:

(1) The frequency of the electrical signal output by the B-TENG corresponds closely to the vibration frequency; once the structural parameters are determined, there exists a constant resonant frequency at which the output electrical signal is significantly enhanced.
(2) Prior to the contact between the charged PTFE ball and the Al electrodes on both sides, the voltage magnitude maintains a good linear relationship with the vibration amplitude. After contact, the magnitude of the characteristic electrical signal between the electrodes remains essentially constant.
(3) In a non-contact state, the mass of the PTFE ball has no effect on its output electrical signal, whereas the length of the polyester rope has a significant impact. Therefore, the appropriate rope length should be chosen based on actual needs.
(4) By analyzing and processing the electrical signals from the B-TENG, the precise detection of vibration amplitude, frequency, and direction can be achieved, with minimal errors of 0.67%, 4.4%, and 5%, respectively. The accuracy of the B-TENG is 0.1 Hz, 0.97 V/mm, 1.5°.

In conclusion, the B-TENG proposed successfully realizes the comprehensive monitoring of vibration amplitude, frequency, and direction. It has the advantages of self-powered, simple structure, and economical manufacturing cost. Considering the advantages, the proposed B-TENG has a great application prospect in the field of marine pipeline vibration state monitoring.

## Figures and Tables

**Table 1:** Comparison of vibration sensors in literature with proposed B-TENG.

Ref.	Working Principle	Structure/Mode	Detection Parameters	SNR/Accuracy
[18]	optical fiber	Fiber Ring Cavity Laser	Frequency: 100–400 kHz	50 dB at 3 kHz
[6]	optical fiber	Single mode–no core–single mode	Frequency: 100–29 kHz	40 dB at 500 Hz
[42]	TENG	Contact-mode freestanding	Frequency: 1–15 Hz Amplitude: 0–16 mm	Not stated
[43]	TENG	Freestanding layer mode	Frequency: 1–8 Hz	Not stated
[44]	TENG	Freestanding layer mode	Frequency: 1–15 Hz Amplitude: 0.5–8 mm	34.5 dB at 10 Hz
This work	TENG	Freestanding layer mode	Frequency: 1–5 Hz Amplitude: 2–14 mm Angle: 0°, 30°, 45°, 60°	0.1 Hz, 0.97 V/mm, and 1.5° at 1–5 Hz

## Data Availability

The data presented in this study are available on request from the corresponding author.

## Supplementary Materials

The following supporting information can be downloaded at: https://www.mdpi.com/article/10.3390/s24123817/s1, Figure S1: Schematic diagram of dynamic analysis of PTFE ball oscillating. Figure S2: Theoretical model of B-TENG, (a) Classical dielectric type B-TENG model. (b) Equivalent circuit model of the B-TENG electrostatic system. Figure S3: Triboelectric series for some commonly materials following a tendency of easy losing electrons (positive) to gaining electrons (negative).
