# Supplementary material for: A Low-Frequency Vibration Sensor Based on Ball Triboelectric Nanogenerator for Marine Pipeline Condition Monitoring
Source: Sensors (Basel). 2024 Jun 13;24(12):3817. doi: 10.3390/s24123817 (PMC11207379; doi:10.3390/s24123817)
Supplement: Supplementary file 1 [file sensors-24-03817-s001.zip › sensors-3028219-supplementary.pdf]

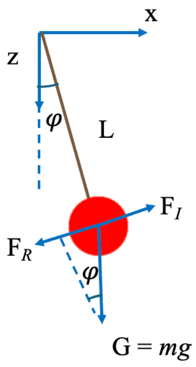

Figure S1. Schematic diagram of dynamic analysis of the PTFE ball oscillating.

The motion of the ball belongs to the secondary transmission, when the ocean pipeline vibration on the acrylic cube, the cube will produce the corresponding transverse rocking, transverse oscillation, and other relative movements, after the forced vibration of the ball is mainly from the cube on the pipeline vibration excitation made by the response. As shown in Fig. S1, when the B-TENG is not affected by external vibration, the inertial force F_I (mass times acceleration) must be equal to the applied action force on the object. In B-TENG, the acting force is the restoring force F_R, caused by the gravitational force G.

$$F_R = -mg\sin\varphi \tag{1}$$

In eq 1, m is the mass of the PTFE ball and g is the acceleration of gravity. The negative sign is caused by the restoring force F_R trying to bring the pendulum back to equilibrium.

Since the inertial force F_I must be expressed in terms of angle φ, this paper assumes that the rope of the pendulum is rigid (its length L is fixed), then the inertial PTFE ball can only move on a circle with radius L. The displacement (spatial coordinate) of the pendulum along this curvature circle is obtained as R'_φ. The angle φ is expressed in radians (180° corresponds to π), so the acceleration can be obtained as:

$$a = \frac{R'd^2\varphi}{dt^2} \tag{2}$$

Thus, according to Newton's law and equation 2, we obtain:

$$\frac{mR'd^2\varphi}{dt^2} = -mg\sin\varphi \tag{3}$$

Simultaneously dividing both sides of the equation by $mR'$and shifting the term on the right side of the equation to the left will give you an equation for a single pendulum unaffected by external driving forces and undamped.

$$\ddot{\varphi} + (\omega_0)^2 sin\varphi = 0 \tag{4}$$

In Eq 4, ω_0 is the angular velocity of the pendulum moving on the curvature circle. However, in practice, the motion inevitably dissipates energy due to damping. Therefore, in this paper, the dissipated energy is also included in the equation of motion by adding a viscous damping term, which is a damping constant with constant velocity, to obtain the equation of motion of the damped pendulum as:

$$\ddot{\varphi} + \gamma\dot{\varphi} + (\omega_0)^2 sin\varphi = 0 \tag{5}$$

where φ is the angle at which the ball swings (angular displacement). ω_0 is the rotational angular velocity of the inertial ball, γ is the damping constant. For a single pendulum motion of a sphere inside a cube, the damping force is given by the Stokes formula with a wall correction factor:

$$F_d = -C_w 6\pi\mu r v \tag{6}$$

Where F_d is the damping force, C_w is the wall correct factor, μ is the kinetic viscosity of the air, r is the radius of the ball, υ is the velocity of the ball relative to the fluid. We assume that the vibration excitation force of the ocean pipeline as a periodic force on the B-TENG, to simplify the model without loss of generality, only the force exerted by the cube on the ball in the horizontal direction is investigated and obtained by analyzing the law of motion in the horizontal direction:

$$\ddot{\varphi} + \gamma\dot{\varphi} + (\omega_0)^2 sin\varphi = -\frac{d^2\left(\frac{x_0}{L}\right)}{dt^2 cos\varphi} \tag{7}$$

$$x_0 = Acos(2\pi f t) \tag{8}$$

Here A and f are the amplitude and frequency, of the external vibration acting on the cube, respectively. Therefore, the law of motion for the forced vibration of the ball can be obtained by adding the damping force with a wall correction factor:

$$\ddot{\varphi} - C_w 6\pi\mu r\dot{\varphi} + (\omega_0)^2 sin\varphi = (2\pi f)^2 \frac{A}{L} cos(2\pi f t) cos\varphi \tag{9}$$

where φ is the angle at which the ball swings (angular displacement), C_w is the wall correct factor. μ is the kinetic viscosity of the air, r is the radius of the ball, $\dot{\varphi}$ is the angular velocity of the ball with respect to the fluid, ω_0 is the rotational angular velocity of the inertial ball, and L is the pendulum length.

For the sine function, the first few terms of its Taylor series expansion are:

$$sin\varphi = \varphi - \frac{\varphi^3}{3!} + \frac{\varphi^5}{5!} - \frac{\varphi^7}{7!} + \cdots \tag{10}$$

The sine function sinφ can be approximated as a linear function using a Taylor series expansion due to the cubic constraints on the motion of the ball and the single pendulum angle φ in the range of the interval -23° to 23°. The sine function can be approximated as:

$$sin\varphi \approx \varphi \tag{11}$$

Under this approximation, the sine function sinφ can be regarded as linear, i.e. approximately equal to its angular value. Similarly, the cosine function can be viewed as linear in a small angular range, approximated as:

$$cos\varphi \approx 1 - \frac{\varphi^2}{2} \tag{12}$$

Thus, under the linear approximation, eq 9 can be simplified to:

$$\ddot{\varphi} - C_w 6\pi\mu r\dot{\varphi} + (\omega_0)^2 \varphi = (2\pi f)^2 \frac{A}{L} cos(2\pi f t) \tag{13}$$

We obtain a linear second order differential equation which describes a linear forced oscillatory system.

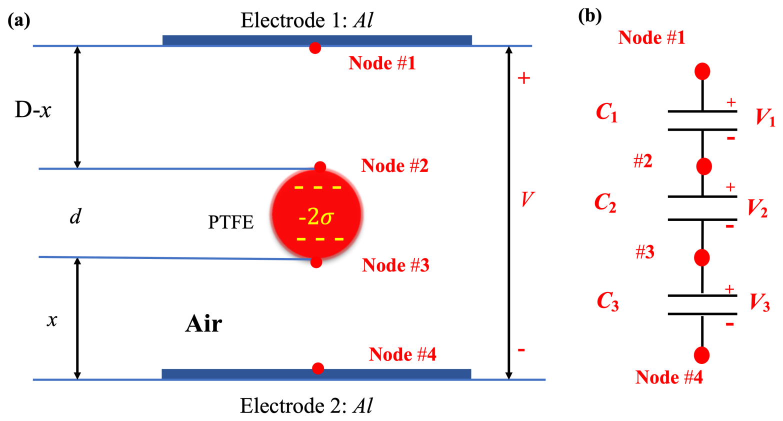

Figure S2. Theoretical model of B-TENG, (a) Classical dielectric type B-TENG model. (b) Equivalent circuit model of the B-TENG electrostatic system.

Fig S2(a) shows a model of B-TENG with a ball diameter of d and a relative dielectric constant of ε_r. The effective dielectric thickness d_0 of the ball is d_1/ε_r. The spacing distance between the two electrodes is D. When the ball meets the top and bottom aluminum electrodes, the surface of the ball is charged due to the contact. For simplicity, assume that the frictional charge of the ball is -σ. Also, due to conservation of charge, the two aluminum electrodes are equally charged with the total amount of positive charge. The equivalent circuit of the B-TENG electrostatic system is shown in Fig S2(b), and we apply the nodal method to analyze this electrostatic system. In practice, the total capacitance between the two electrodes (node 1 and node 4) is a series connection of C_1, C_2, and C_3, which is expressed by the following equation.

$$C = \frac{1}{\frac{1}{C_1} + \frac{1}{C_2} + \frac{1}{C_3}} = \frac{\varepsilon_0 + S}{d_0 + D} \tag{14}$$

Firstly, we analyze the charge distribution in the short-circuit case (node 1 and node 4 have the same potential) by setting the total charge of the ball to be $2\sigma S$, The total charge at node 1 is Q_1, and the total charge at node 4 is $Q_2 = 2\sigma S - Q_1$. According to the basic electrodynamics and the principle of conservation of charge, we can obtain:

$$Q_1 = \sigma S \frac{\frac{1}{C_2} + \frac{2}{C_3}}{\frac{1}{C_1} + \frac{1}{C_2} + \frac{1}{C_3}} \tag{15}$$

$$Q_2 = \sigma S \frac{\frac{2}{C_1} + \frac{1}{C_2}}{\frac{1}{C_1} + \frac{1}{C_2} + \frac{1}{C_3}} \tag{16}$$

In practical applications, where the effective dielectric thickness of the dielectric is often negligible compared to the air gap, Eq 15 and 16 further simplify to:

$$Q_1 \approx \sigma S \frac{\frac{1}{C_3}}{\frac{1}{C_1} + \frac{1}{C_3}} \tag{17}$$

$$Q_2 \approx \frac{2\sigma S}{1+\frac{C_1}{C_3}} \tag{18}$$

According to equations 17 and 18, the working principle of B-TENG can be obtained. When x = 0, C_3 is infinite, so Q_1 is close to 0 and Q_2 is approximately equal to 2σS. In this condition, all positive friction charges are attracted to the bottom electrode by the negative charges on the surface of the ball. Similarly, when x = D, all positive charges are attracted to the top electrode. Thus, a change in the ball position can cause a change in the capacitance ratio of C_1/C_3, driving electrons to flow between the two electrodes in the case of a short circuit. Under the minimum achievable charge reference state (MACRS), the short circuit transferred charges (Q) and open-circuit voltage (V_{OC}) can be calculated as follows:

$$Q = \frac{2\sigma x}{d_0 + D} \tag{19}$$

$$V_{OC} = \frac{2\sigma x}{\varepsilon_0} \tag{20}$$

The governing equation of B-TENG can be written as:

$$V = -\frac{1}{C}Q + V_{OC} = -\frac{d_0 + D}{\varepsilon_0 S}Q + \frac{2\sigma x}{\varepsilon_0} \tag{21}$$

For a detailed derivation of the above equations refer to [1].

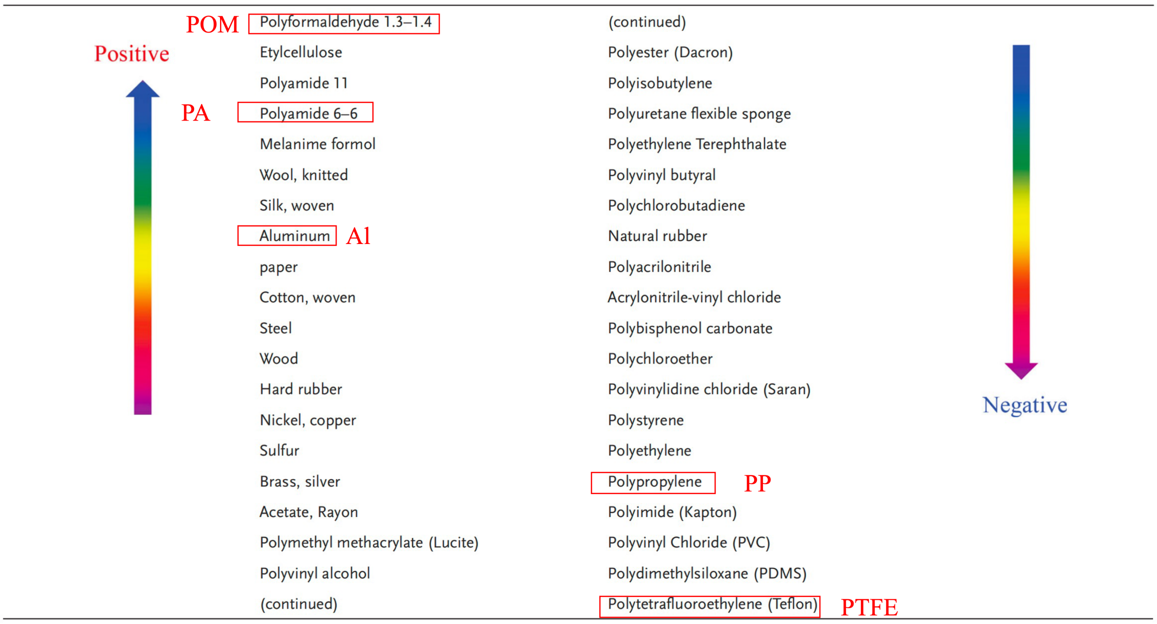

Figure S3. Triboelectric series for some commonly materials following a tendency of easy losing electrons (positive) to gaining electrons (negative) [from ref. 2].

According to the triboelectric series in ref. [2], consider cost and accessibility, a variety of common friction materials such as polyformaldehyde (POM), polypropylene (PP), nylon (PA) and polytetrafluoroethylene (PTFE) were selected for comparative analysis.

1. Niu, S.; Liu, Y.; Chen, X.; Wang, S.; Zhou, Y.S.; Lin, L.; Xie, Y.; Wang, Z.L. Theory of freestanding triboelectric-layer-based nanogenerators. *Nano Energy* **2015**, *12*, 760-774, http://doi.org/10.1016/j.nanoen.2015.01.013.
2. Zhang, C.; Tang, W.; Han, C.; Fan, F.; Wang, Z.L. Theoretical Comparison, Equivalent Transformation, and Conjunction Operations of Electromagnetic Induction Generator and Triboelectric Nanogenerator for Harvesting Mechanical Energy. *Adv. Mater.* **2014**, *26*, 3580-3591, http://doi.org/https://doi.org/10.1002/adma.201400207.
